# Diabetic hand infections: a case series highlighting diagnostic and therapeutic challenges

**DOI:** 10.1186/s12879-026-14076-x

**Published:** 2026-07-24

**Authors:** Meryem Billur Miroğlu, Sibel Yıldız Kaya, Rıdvan Karaali, Gözde Dalgan, Çağlar Yılmaz, Berrak Karatan, Neşe Saltoğlu

**Affiliations:** 1 Cerrahpaşa Medical Faculty, Department of Infectious Diseases and Clinical Microbiology, Istanbul University-Cerrahpaşa, Istanbul, Türkiye; 2 Cerrahpaşa Medical Faculty, Department of Plastic, Reconstructive and Aesthetic Surgery, Istanbul University-Cerrahpaşa, Istanbul, Türkiye

**Keywords:** Diabetic hand infection, Diabetes mellitus, Osteomyelitis, Polymicrobial infection, Amputation, Surgical treatment

## Abstract

**Background:**

Diabetic hand infections are uncommon but potentially severe complications of diabetes mellitus. Because of the complex anatomy and functional importance of the hand, these infections may progress rapidly and result in serious morbidity, including osteomyelitis and amputation. Clinical data on this condition remain limited.

**Methods:**

This case series included patients hospitalized with diabetic hand infection at a tertiary care center between 2020 and 2026. Patients, ≥ 18 year-old, with confirmed diabetes mellitus and clinical, microbiological, and/or radiological evidence of hand infection were included. Demographic data, comorbidities, HbA1c levels, laboratory findings, microbiological results, imaging findings, and treatment outcomes were reviewed from medical records.

**Results:**

Seven patients were included. Osteomyelitis was identified in 57% of patients, while 43% had isolated soft tissue infection. Polymicrobial infection was present in 85% of cases. *Staphylococcus* spp. were the most commonly isolated pathogens, followed by *Enterococcus* spp. and Gram-negative organisms. Gram-negative bacteria were identified in 28% of cases. Surgical intervention was required in most patients, and 28% underwent amputation. Poor glycemic control, minor trauma, polymicrobial infection, and deep tissue involvement were common features in this series.

**Conclusions:**

Diabetic hand infections should be recognized as serious and potentially aggressive complications of diabetes mellitus. Early diagnosis, appropriate imaging, timely surgical management, and culture-guided antimicrobial therapy are essential to improve outcomes. Larger multicenter studies are needed to better define prognostic factors and to guide more standardized treatment strategies.

## Background

Diabetes mellitus (DM) is associated with impaired immune responses, microvascular abnormalities, and delayed wound healing, all of which increase susceptibility to infection. In addition, chronic hyperglycemia impairs neutrophil functions such as chemotaxis, phagocytosis, and intracellular killing, resulting in reduced host defense mechanisms [1, 2].

Although infections are common in patients with diabetes, most published studies have focused on lower extremity infections. In contrast, diabetic hand infections are relatively uncommon and remain less clearly defined in the literature [3, 4]. Despite being less frequent, these infections may cause serious functional impairment because of the complex anatomy and essential function of the hand. The reported incidence of diabetic hand infections ranges from 0.7% to 12% [5]. These infections may show a rapid and aggressive course, progressing to deep tissue involvement, osteomyelitis, gangrene, and even limb loss [5].

Minor trauma is often described as a precipitating factor. Small cuts, puncture wounds, and foreign body injuries may create a portal of entry for microorganisms. In tropical regions, this condition has been termed “tropical diabetic hand syndrome,” which is characterized by rapidly progressive cellulitis, abscess formation, and tissue necrosis following trivial trauma [6, 7]. The microbiological spectrum is broad. Gram-positive bacteria, particularly Staphylococcus aureus, are the most commonly reported pathogens; however, gram-negative bacteria and opportunistic microorganisms may also be isolated, especially in complicated cases [8, 9].

Since the available literature is limited, further clinical data are needed to better define the features and outcomes of diabetic hand infections. In this study, we aimed to evaluate the clinical characteristics, microbiological spectrum, and treatment outcomes of patients managed at a tertiary care center.

## Methods

This case series included patients hospitalized with diabetic hand infection at Cerrahpaşa Medical Faculty between 2020 and 2026. Patients aged 18 years or older with confirmed diabetes mellitus and clinical, microbiological, and/or radiological findings consistent with hand infection were included. Patients aged < 18 years were excluded. Demographic characteristics, comorbidities, HbA1c (glycated hemoglobin A1c) levels, laboratory findings, microbiological culture results, imaging findings, and treatment data were obtained from medical records. Radiological evaluation was performed using plain radiography and/or magnetic resonance imaging (MRI). Empirical antibiotic therapy was initiated at presentation and subsequently adjusted according to culture results. Surgical interventions, including debridement, abscess drainage, and amputation, were performed when clinically indicated.

### Descriptive statistics were used to summarize the data

The study was conducted in accordance with the Declaration of Helsinki and was approved by the Cerrahpaşa Faculty of Medicine Medical Research Ethics Committee (approval number: dT23ef0l). The datasets generated and analysed during the current study are not publicly available due to patient confidentiality and ethical restrictions but are available from the corresponding author on reasonable request. No financial support was received for the conduct of this study.

### Case presentations

#### Case 1

**Fig. 1:**
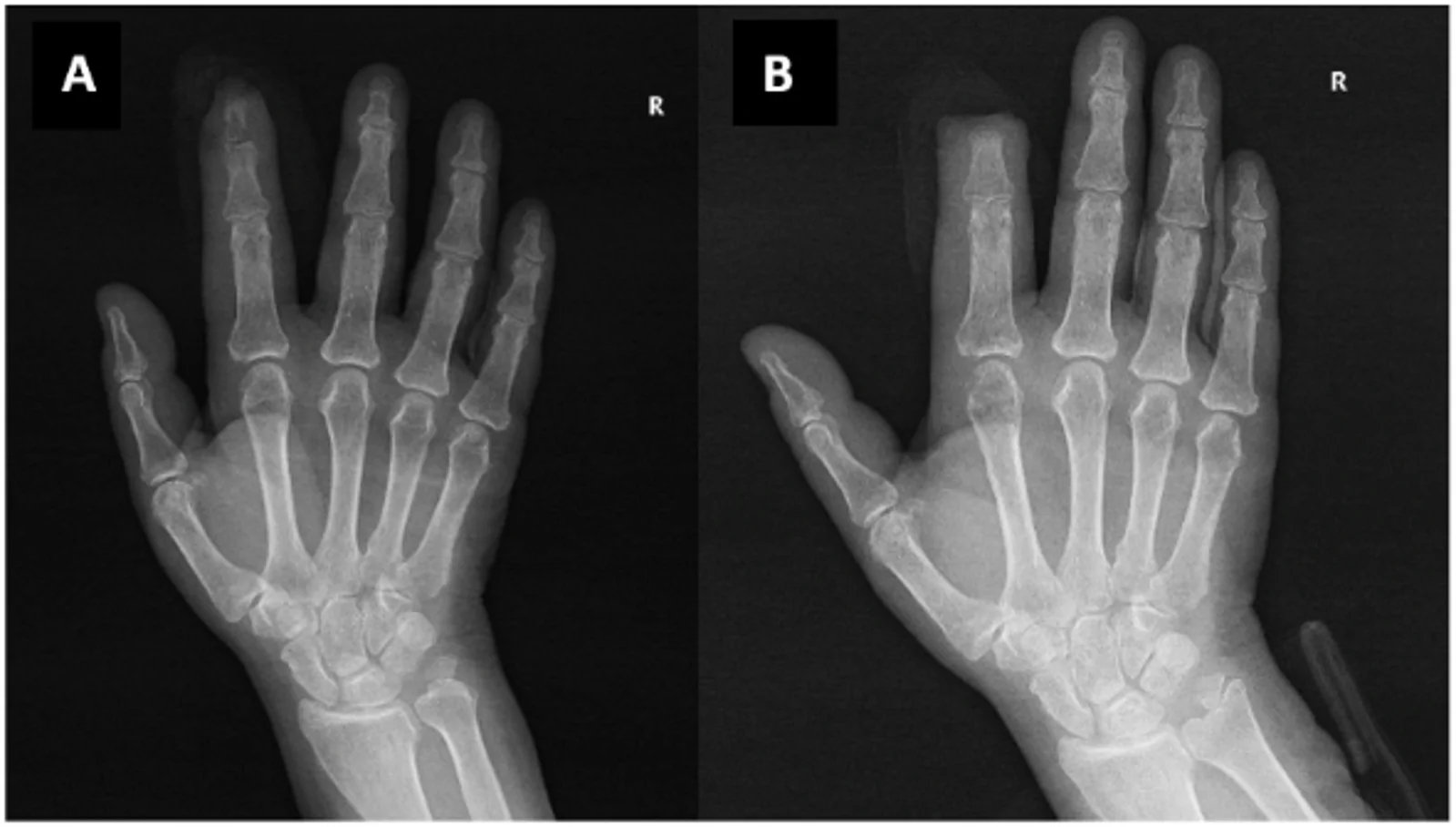
Pre-amputation (**A**) and post-amputation (**B**) plain radiograph of Case 1

**Fig. 2:**
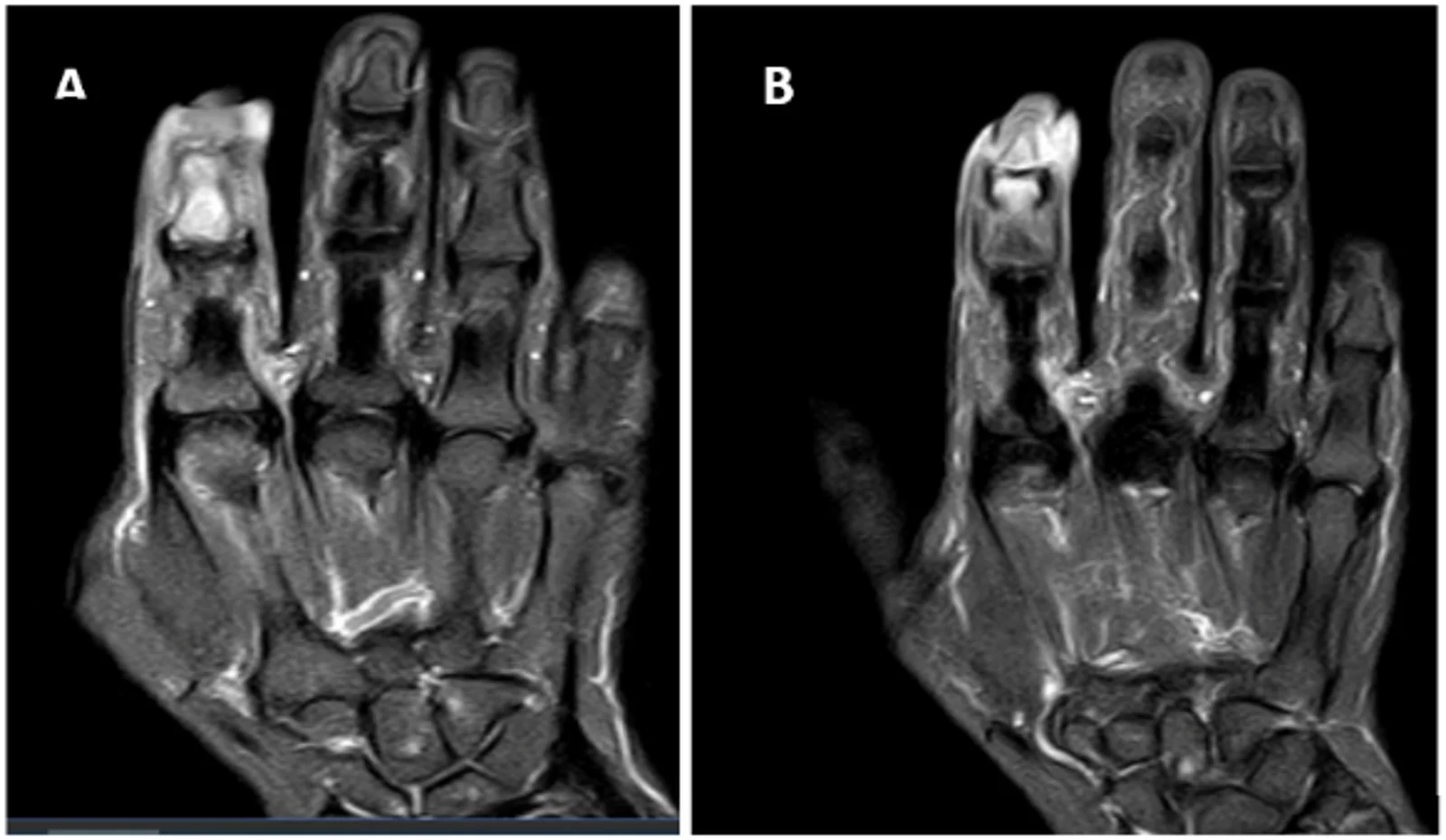
Magnetic resonance imaging of Case 1 demonstrating findings consistent with osteomyelitis

A 55-year-old woman with an 11-year history of type 2 diabetes mellitus and hypertension presented with swelling and erythema of the right second finger. Her HbA1c level was 12.9%. Incision and drainage were performed, and tissue cultures yielded Enterococcus spp. and methicillin-resistant coagulase-negative *Staphylococcus* spp. MRI demonstrated osteomyelitis (Figs. 1 and 2). Although teicoplanin and piperacillin–tazobactam were administered, distal phalanx amputation was required. The patient showed clinical and radiological improvement during follow-up.

#### Case 2

**Fig. 3:**
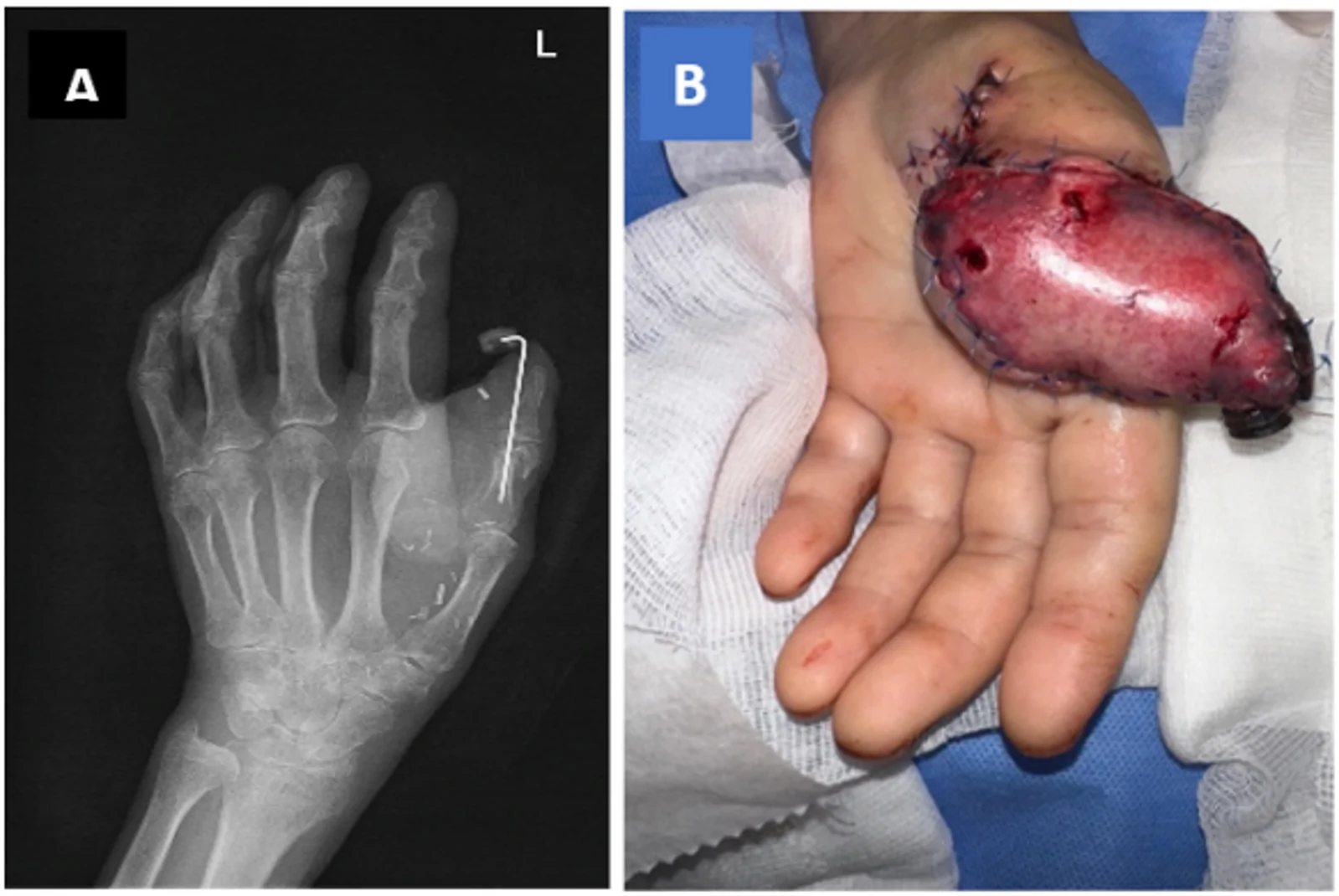
Plain radiograph (**A**) and clinical photograph of the hand of Case 2

A 63-year-old woman was admitted with a complicated soft tissue infection of the left thumb that developed after trauma. She had a history of diabetes mellitus, hypertension, and hyperlipidemia. Cultures yielded methicillin-resistant *Staphylococcus aureus*,* Corynebacterium* spp., and *Candida* spp. Multiple surgical interventions, including debridement and grafting, were required (Fig. 3). She received prolonged antimicrobial therapy, with eventual clinical resolution.

#### Case 3

**Fig. 4:**
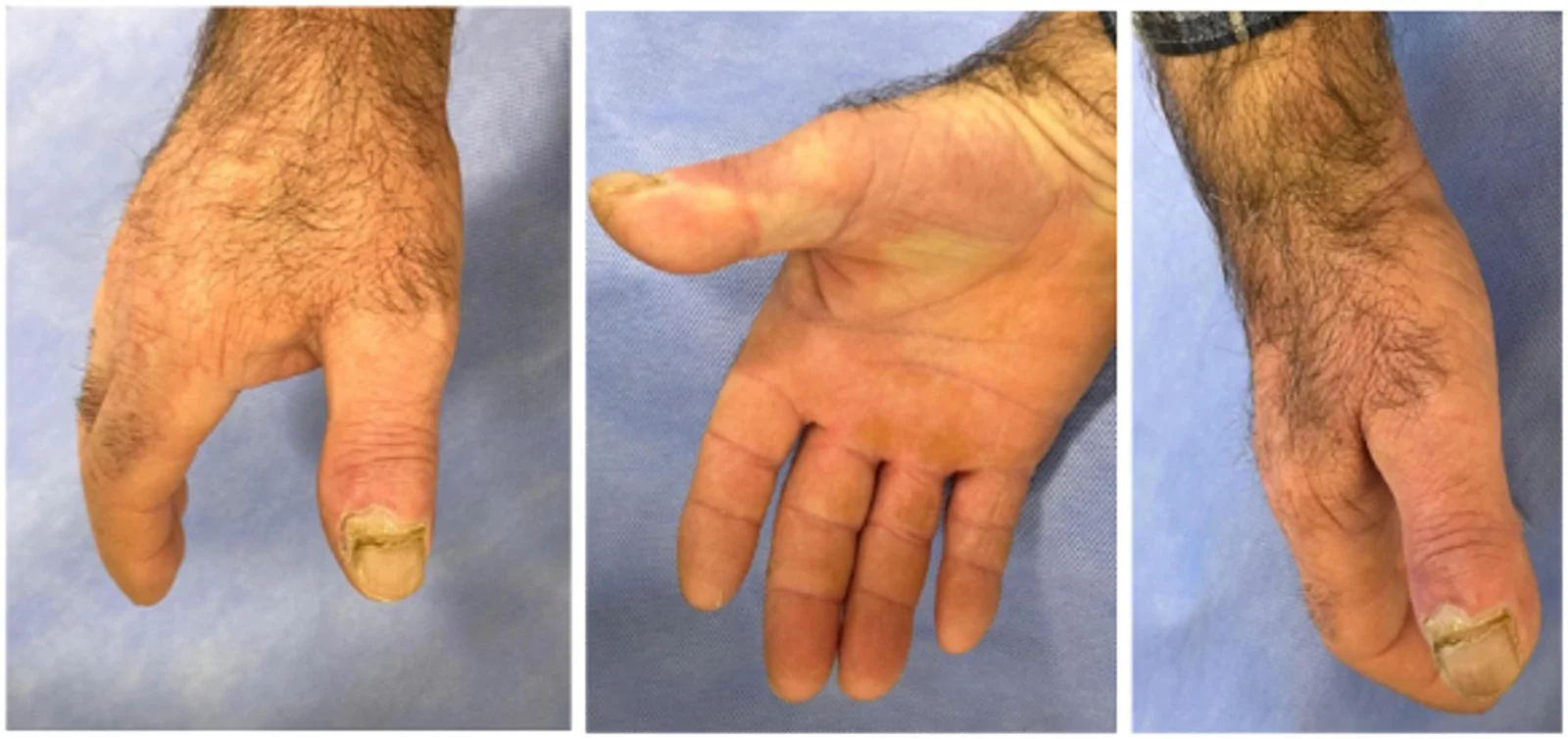
Clinical photographs of the hand of Case 3 from different perspectives

**Fig. 5:**
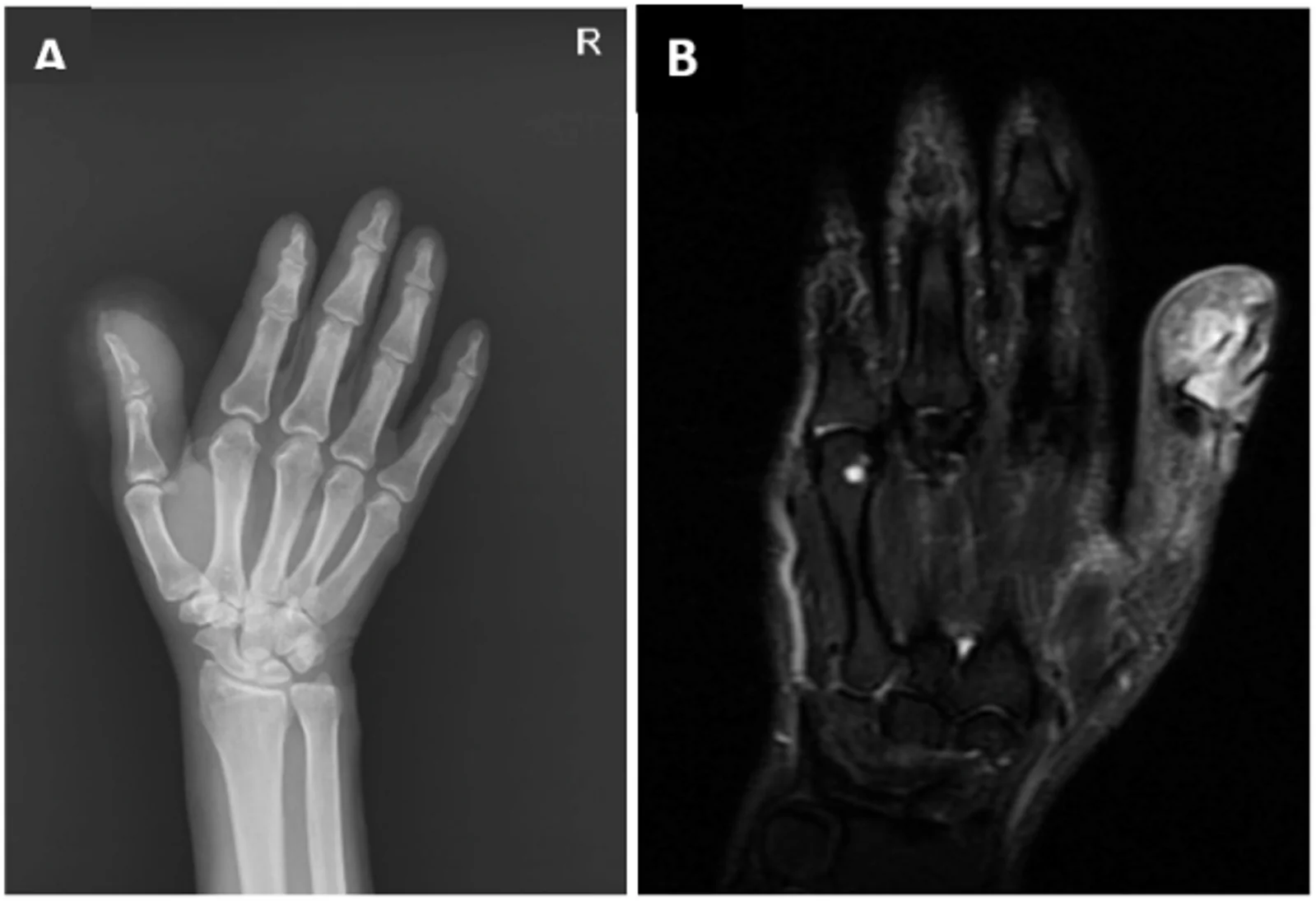
Plain radiograph (**A**) and magnetic resonance imaging (**B**) of Case 3 demonstrating findings consistent with acute osteomyelitis

A 59-year-old man presented with hand infection after a fishbone injury (Fig. 4). Initial outpatient treatment failed to control the infection. MRI demonstrated osteomyelitis (Fig. 5), and culture yielded *Enterococcus* spp. He was treated with several antibiotic regimens and improved after 6 weeks of therapy.

#### Case 4

**Fig. 6:**
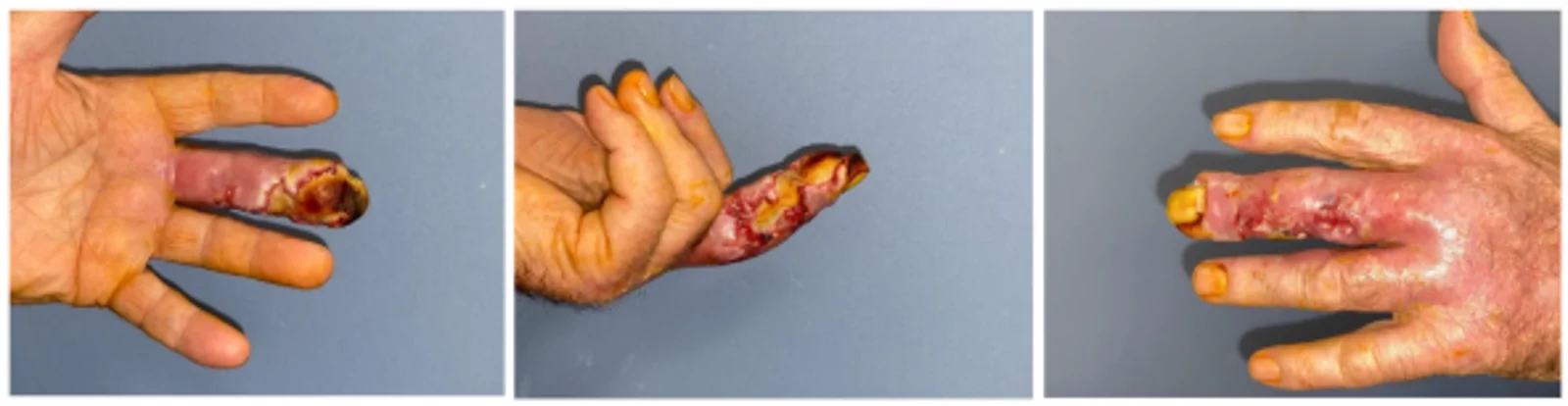
Clinical photographs of the hand of Case 4 from different perspectives

**Fig. 7:**
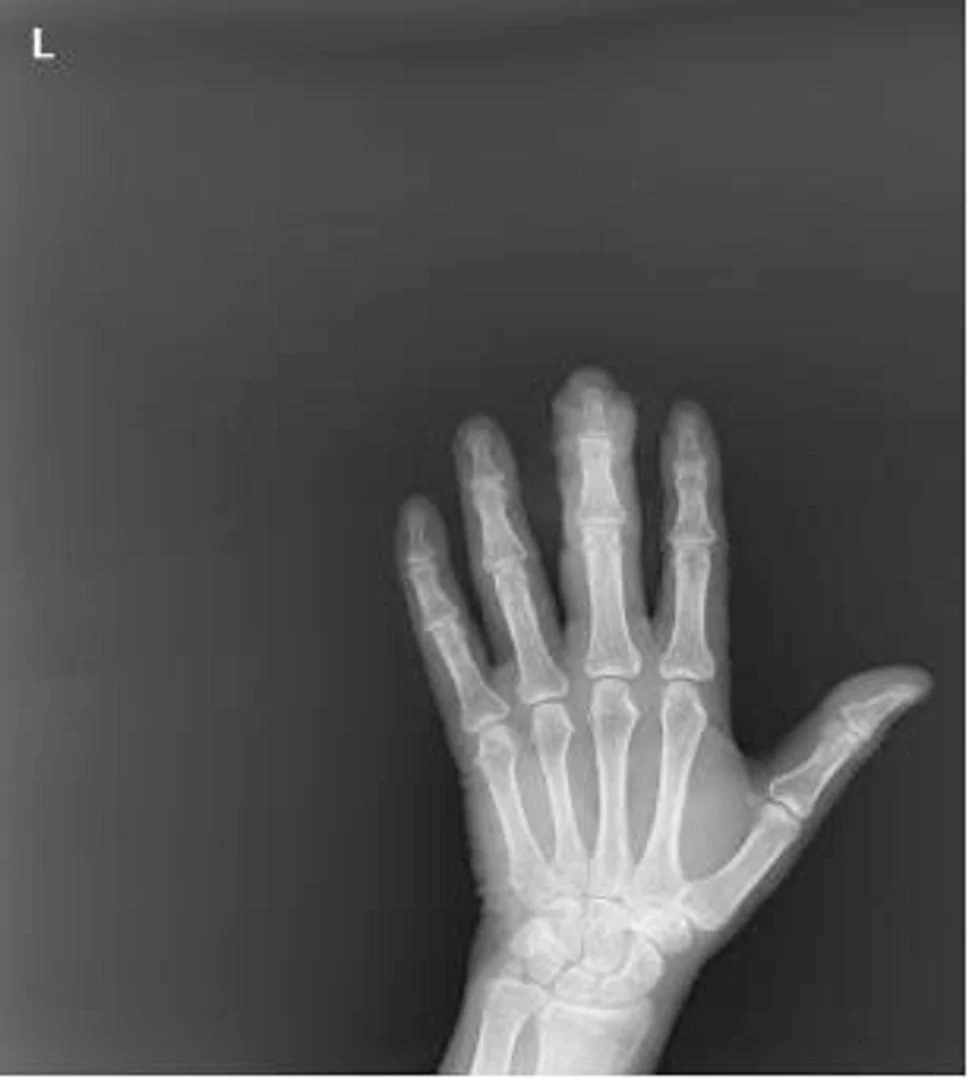
Plain radiograph of Case 4

A 79-year-old man was admitted with a necrotizing infection of the third finger (Fig. 6). Cultures yielded a polymicrobial gram-negative infection. As the infection progressed to osteomyelitis (Fig. 7), amputation at the proximal interphalangeal level was performed.

#### Case 5

**Fig. 8:**
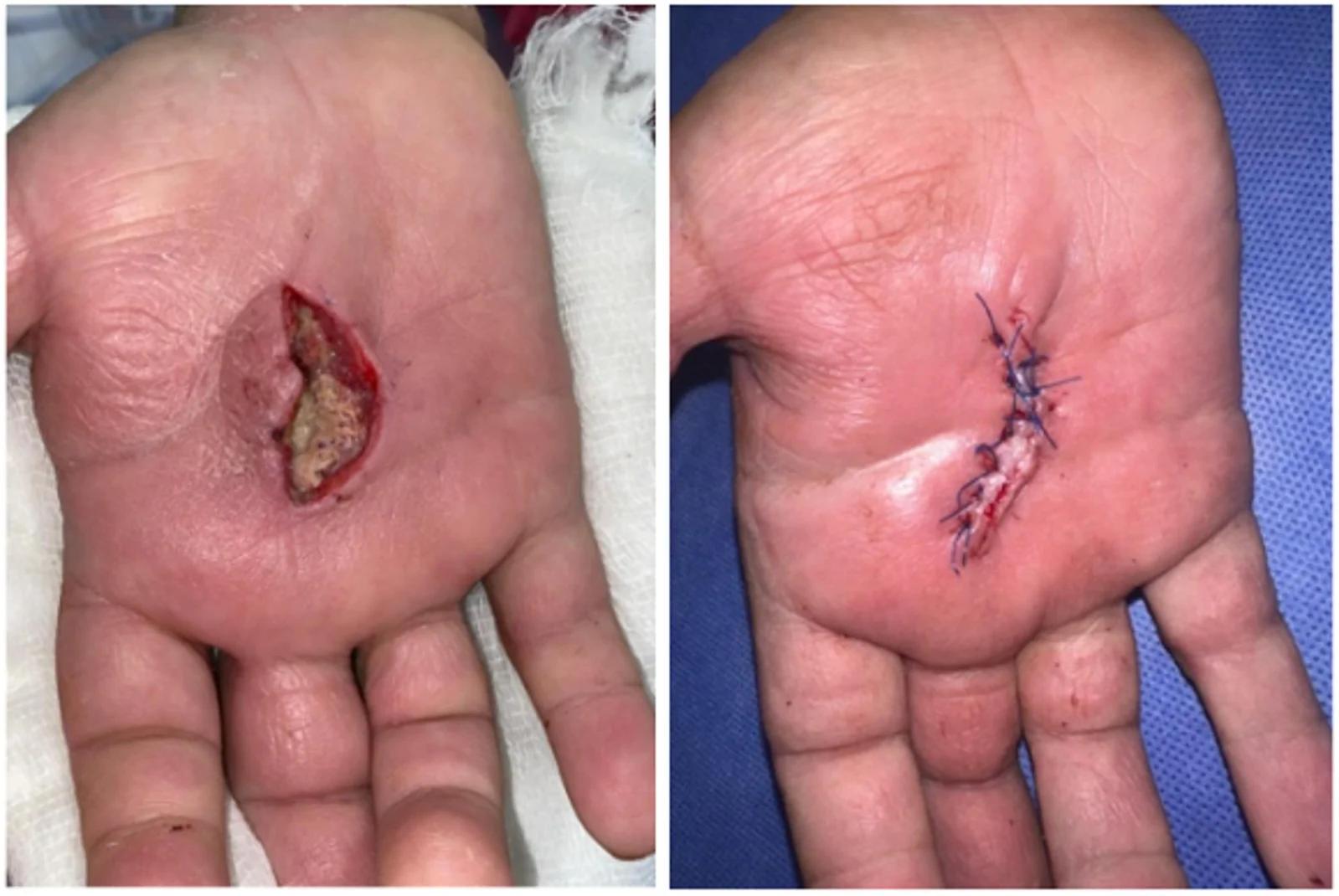
Clinical photographs of the hand of Case 5 from different perspectives

**Fig. 9:**
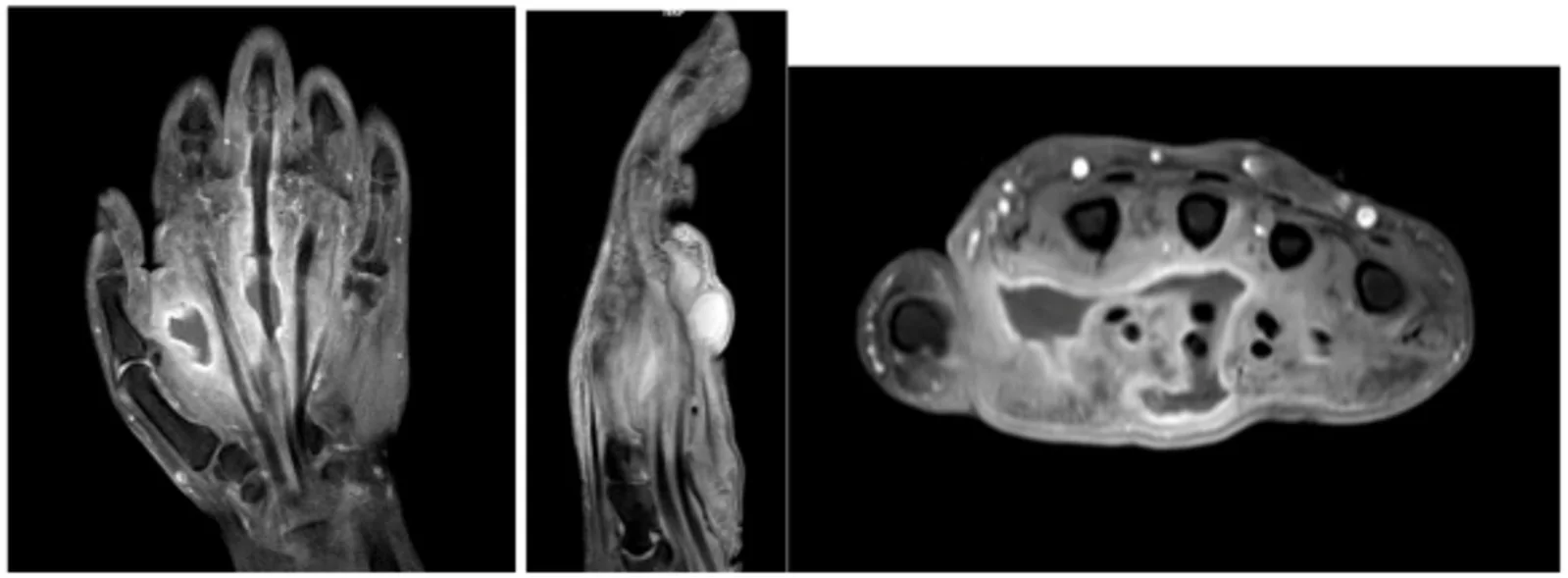
Magnetic resonance imaging of Case 5 demonstrating findings consistent with abscess

A 53-year-old woman was admitted with cellulitis and abscess formation (Fig. 8). MRI revealed an abscess without associated osteomyelitis (Fig. 9). Microbiological evaluation showed a polymicrobial infection, including resistant organisms. She required repeated debridement and treatment with broad-spectrum antibiotics.

#### Case 6

**Fig. 10:**
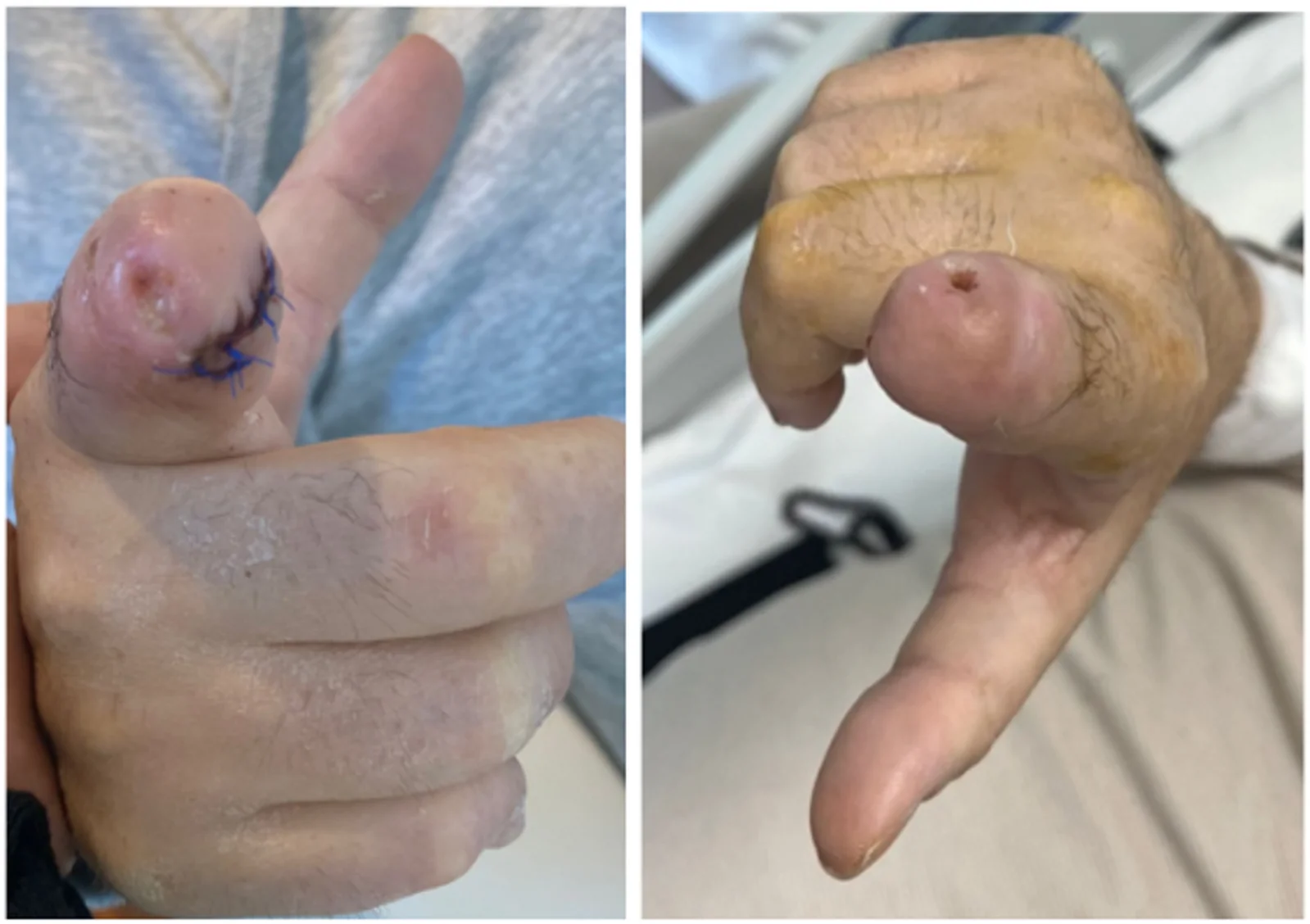
Clinical photographs of the hand of Case 6 from different perspectives

**Fig. 11:**
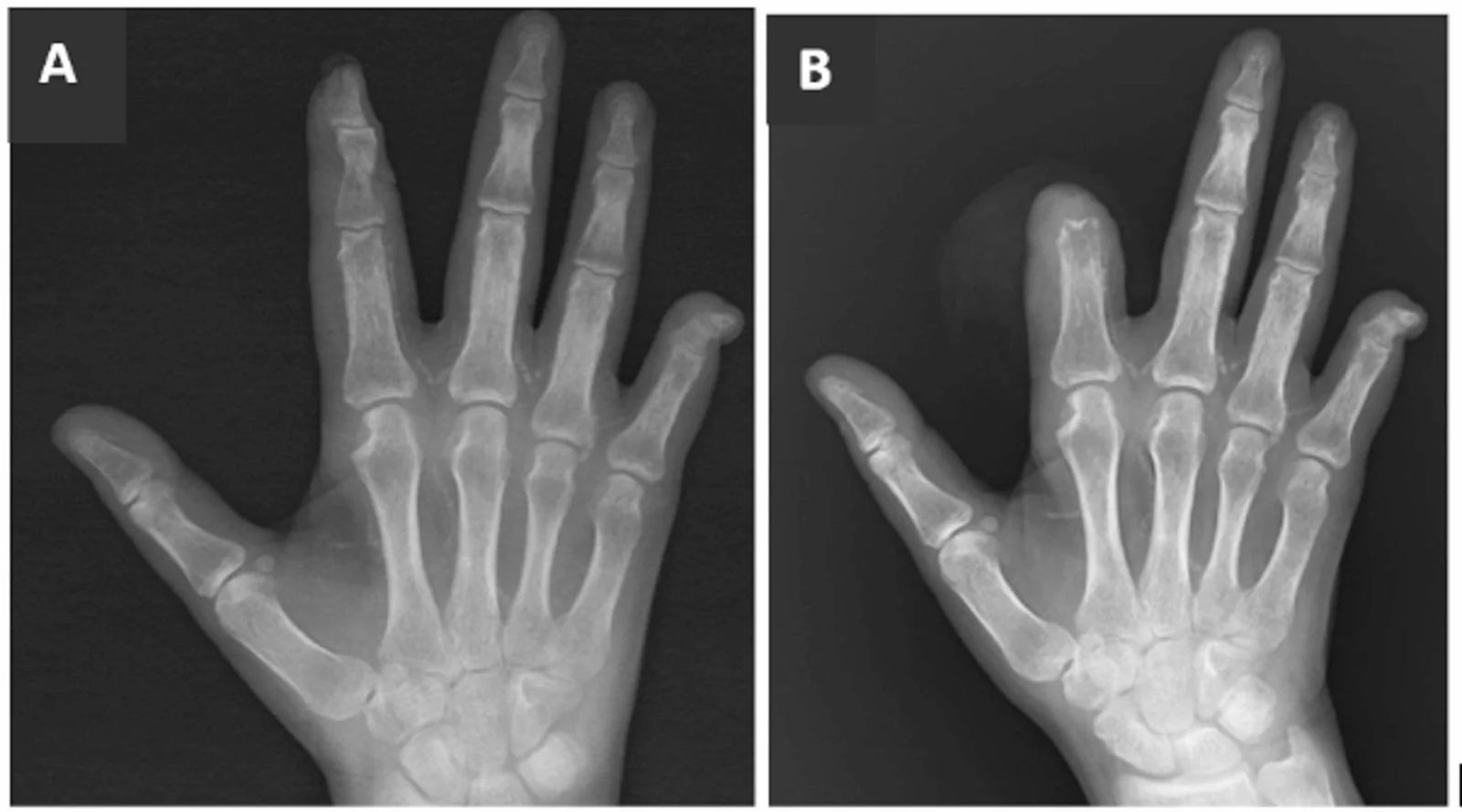
Pre-amputation (**A**) and post-amputation (**B**) plain radiograph of Case 6

A 49-year-old man with multiple comorbidities presented with a postoperative infection following a previous amputation (Fig. 10). Microbiological cultures grew methicillin-resistant *Staphylococcus aureus* (MRSA) and *Streptococcus agalactiae*. Because of chronic osteomyelitis (Fig. 11), prolonged antibiotic treatment was required.

#### Case 7

**Fig. 12:**
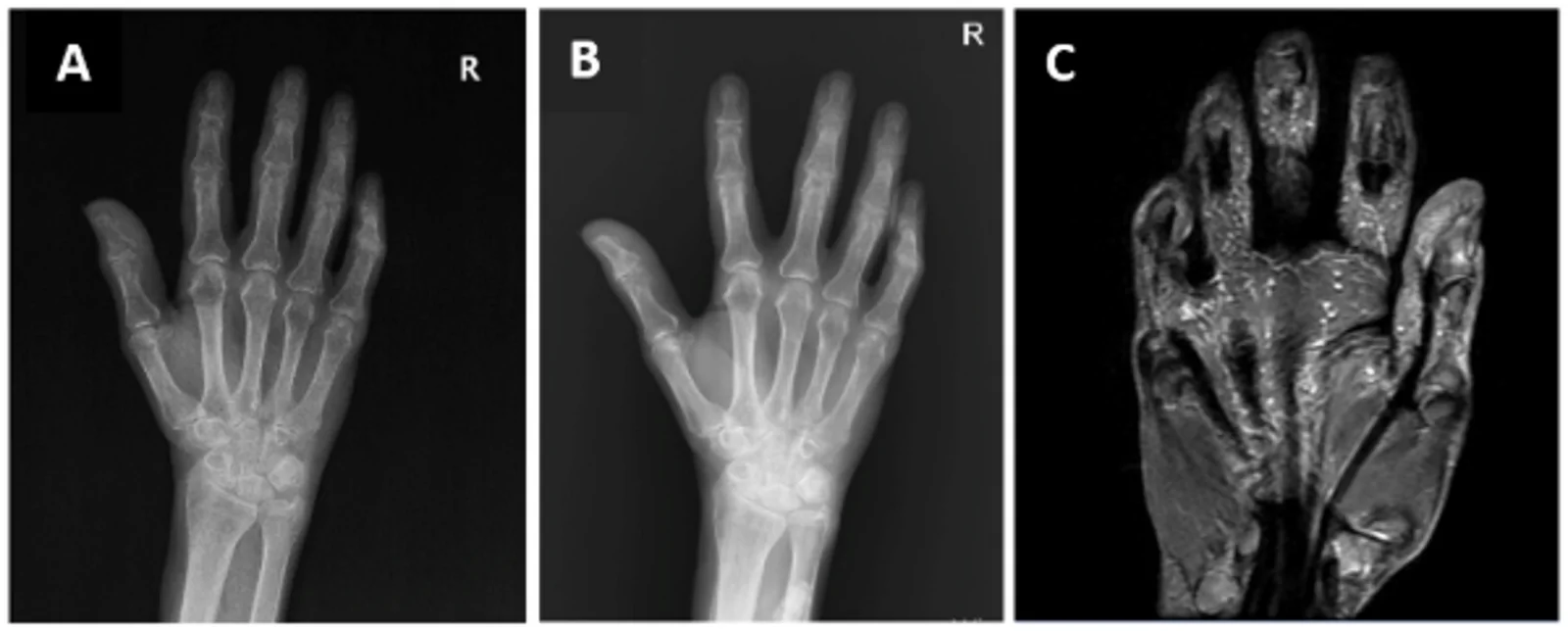
Plain radiographs (**A**-**B**) and magnetic resonance imaging (**C**) of Case 7

A 73-year-old woman was admitted with swelling of the thumb. Initial outpatient treatment failed to control the infection. MRI demonstrated osteomyelitis (Fig. 12), and cultures grew *Staphylococcus lugdunensis* and *Klebsiella* spp. She responded well to surgical drainage and prolonged antibiotic treatment (Tables 1,2 and 3).

## Results

Seven patients were included in the study. Their demographic characteristics, comorbidities, and antidiabetic treatments are summarized in Table 1. Baseline laboratory parameters at presentation are presented in Table 2. Osteomyelitis was identified in 57% of patients, while 43% had isolated soft tissue infection. Polymicrobial infection was present in 85% of cases. *Staphylococcus* spp. were the most common pathogens, followed by *Enterococcus* spp. and Gram-negative organisms. Gram-negative bacteria were identified in 28% of cases. Surgical intervention was required in 86% of patients, and 28% underwent amputation. The clinical features, microbiological findings, and treatment outcomes are detailed in Table 3.

**Table 1:** Patient demographics, comorbidities, and antidiabetic treatment

Case	Age (years)	Sex	Comorbidities	Oral Antidiabetic Therapy	Insulin Therapy
1	55	Female	Hypertension	Yes	Yes
2	63	Female	Hypertension, Hyperlipidemia	Yes	Yes
3	59	Male	None	Yes	No
4	79	Male	Hypertension	Yes	Yes
5	53	Female	Hypertension, Psoriasis, Bullous Pemphigoid	Yes	Yes
6	49	Male	Hypertension, Coronary Artery Disease, Chronic Kidney Disease	Yes	Yes
7	73	Female	Hypertension, Hashimoto Thyroiditis	Yes	Yes

**Table 2:** Baseline laboratory parameters at presentation

Case	WBC (×10³/µL)	Neutrophils (%)	CRP (mg/dL)	ESR (mm/h)	HbA1c (%)	Urea / Creatinine (mg/dL)
**1**	7.2	49.2	2.46	8	12.9	24 / 0.74
**2**	10.8	61.0	2.39	30	7.6	55 / 0.93
**3**	7.91	61.0	4.9	41	8.8	37 / 0.85
**4**	9.91	69.7	2.85	32	11.9	60 / 1.22
**5**	7.08	61.3	50	102	8.6	46 / 0.86
**6**	12.53	81.8	158	44	8.9	58 / 3.57
**7**	12.78	72.1	3	31	7.4	43 / 0.77

**Table 3:** Clinical features, microbiological findings, and treatment outcomes

Case	Clinical Presentation	Pathogens	Wound Care	Surgical Management	Antibiotic Therapy	Treatment Duration
**1**	Paronychia + osteomyelitis	*Enterococcus spp.*, MR-CoNS	Polyhexanide + poloxamer gel/spray	Distal phalanx amputation	Piperacillin–tazobactam, teicoplanin	26 days
**2**	Complicated soft tissue infection	MRSA, *Corynebacterium spp.*, *Candida spp.*	Normal saline	Debridement + grafting	Ceftriaxone, teicoplanin, fluconazole, linezolid	7 weeks
**3**	Osteomyelitis	*Enterococcus spp.*	Normal saline	Debridement	Piperacillin–tazobactam, teicoplanin, linezolid, amoxicillin–clavulanate, ciprofloxacin	6 weeks
**4**	Necrotizing infection + osteomyelitis	*Proteus mirabilis*, *Escherichia coli*, *Morganella morganii*	Hypochlorous acid solution	PIP joint amputation	Piperacillin–tazobactam, teicoplanin, cefepime, ciprofloxacin	3 weeks
**5**	Cellulitis + abscess	*Corynebacterium spp.*, MR-CoNS	Hypochlorous acid solution	Debridement	Amoxicillin–clavulanate, ciprofloxacin, meropenem, tigecycline, teicoplanin	25 days
**6**	Postoperative infection	MRSA, *Streptococcus agalactiae*	Hypochlorous acid solution	Debridement	Ciprofloxacin, piperacillin–tazobactam, teicoplanin	90 days
**7**	Osteomyelitis	*Staphylococcus lugdunensis*, *Klebsiella spp.*	Normal saline	Abscess drainage	Amoxicillin–clavulanate, ciprofloxacin, clindamycin, ertapenem	3 months

## Discussion

Diabetic hand infections remain a relatively under-recognized complication within the broad spectrum of diabetes-related infections. However, despite being less common than diabetic foot infections, they may lead to substantial morbidity because of the complex anatomy and critical functional role of the hand. Their clinical course is often aggressive, with rapid progression to deep tissue involvement and a considerable risk of amputation [3–5]. Therefore, their low frequency should not lead to underestimation, since delayed diagnosis and treatment may result in serious functional loss and permanent disability.

In our series, the median age of the patients was 59 years (range: 49–79 years), which is consistent with previous reports showing that diabetic hand infections are more common in middle-aged and elderly individuals [3–5]. Poor glycemic control was also a notable finding, HbA1c levels were above 7% in all patients and above 10% in two patients. This observation is consistent with previous studies and suggests that inadequate glycemic control may increase both the risk and severity of infection in patients with diabetic hand involvement [1, 10, 11]. Hyperglycemia is known to impair several components of the immune response, including neutrophil chemotaxis, phagocytosis, and intracellular killing, thereby weakening host defense and facilitating the progression of infection [1, 2, 10].

Another important finding in our study was the important role of minor trauma as a triggering factor. Even small injuries appeared sufficient to initiate infection, emphasizing the vulnerability of patients with diabetes to apparently minor skin or soft tissue damage. This pattern is similar to the concept of “tropical diabetic hand syndrome,” in which minor trauma may be followed by rapidly progressive cellulitis, abscess formation, and tissue necrosis [6, 7]. However, these findings suggest that this phenomenon is not restricted to tropical settings but may represent a broader pathophysiological pattern applicable across diverse populations. From a clinical perspective, this point is important because it highlights the need for careful evaluation and early treatment even after minor hand injuries in patients with diabetes.

From a microbiological perspective, our findings support the heterogeneous nature of diabetic hand infections. In our series, polymicrobial infection was observed in 85% of cases, which is in agreement with earlier reports describing the mixed microbiological profile of these infections [8]. *Staphylococcus* spp. were the most commonly isolated pathogens, but gram-negative organisms were also identified in 28% of patients. This finding supports previous observations suggesting that higher prevalence of gram-negative organisms in complicated infections [9]. The presence of resistant and gram-negative pathogens may complicate treatment; therefore, local microbiological patterns and individual patient characteristics should be considered when selecting initial empirical antimicrobial therapy [12, 13].

Surgical treatment remains a major component of management in diabetic hand infections. Most patients in our study underwent surgical intervention, and the amputation rate was 28%, which appears comparable to rates reported in the literature [5, 14]. This high rate of surgery reflects the aggressive nature of these infections and the limited role of conservative treatment alone in advanced cases. Early debridement and timely drainage are essential for infection control and for preventing further tissue destruction [15]. Even when amputation involves only part of a digit, the functional consequences may still be substantial.

The high frequency of osteomyelitis in our series further highlights the severity of diabetic hand infections. Bone involvement is a well-recognized marker of poor outcome and has been associated with an increased risk of digital amputation [11, 14–16]. Our findings are consistent with this observation and support the importance of early imaging and prompt treatment when deep infection is suspected.

Diabetic hand infections may also have important long-term consequences beyond the acute phase. Even digital amputation has been associated with reduced quality of life and increased mortality in previous studies [17, 18]. In addition, some patients may require further procedures or revision surgery after initial treatment. These findings show that management should focus not only on infection control, but also on preservation of function, rehabilitation, and long-term follow-up.

This study has several limitations. First, it was retrospective and included a small number of patients. Second, because of its descriptive design, it does not allow the identification of independent predictors of outcome. Nevertheless, given the relative rarity of diabetic hand infections, our findings still provide useful clinical and microbiological information. They also highlight the need for larger multicenter studies to better define prognostic factors and to support more standardized treatment strategies.

## Conclusion

Diabetic hand infections should be regarded as a distinct and clinically important complication of diabetes mellitus. Early recognition, appropriate imaging, timely surgical intervention, and culture-guided antimicrobial therapy are essential for improving outcomes. Further studies are needed to optimize management strategies and to identify preventive approaches in high-risk patients.

## Data Availability

The datasets generated and analysed during the current study are not publicly available due to patient confidentiality and ethical restrictions but are available from the corresponding author on reasonable request.

## Abbreviations

CRP: C-reactive protein
DM: Diabetes mellitus
ESR: Erythrocyte sedimentation rate
HbA1c: Glycated hemoglobin A1c
MR-CoNS: Methicillin-resistant coagulase-negative staphylococci
MRI: Magnetic resonance imaging
MRSA: Methicillin-resistant staphylococcus aureus
PIP: Proximal interphalangeal
WBC: White blood cell count
